# Robotic prostatectomy after abandoned open radical prostatectomy—Technical aspects and outcomes

**DOI:** 10.1002/bco2.34

**Published:** 2020-08-30

**Authors:** E. O’Connor, S. Koschel, D. Bagguley, N. J. Sathianathen, M. G. Cumberbatch, I. A. Thangasamy, D. Moon, D. G. Murphy

**Affiliations:** ^1^ Division of Cancer Surgery Peter MacCallum Cancer Centre Melbourne VIC Australia; ^2^ Department of Surgery University of Melbourne Austin Hospital Heidelberg VIC Australia; ^3^ EJ Whitten Prostate Cancer Research Centre at Epworth Melbourne VIC Australia; ^4^ Department of Urology Northern Health Melbourne VIC Australia; ^5^ Department of Academic Urology Royal Hallamshire Hospital Sheffield UK; ^6^ Faculty of Medicine University of Queensland Brisbane QLD Australia; ^7^ Sir Peter MacCallum Department of Oncology University of Melbourne Parkville VIC Australia

**Keywords:** complications, prostatectomy, prostate cancer, reoperation, robotic surgical procedures

## Abstract

**Objective:**

To describe the technical aspects and outcomes of robotic‐assisted radical prostatectomy (RARP) following abandoned open radical prostatectomy (ORP).

**Patients and Methods:**

A retrospective review was performed of patients who underwent RARP following abandonment of ORP between 2016 and 2020. RARP was undertaken by two highly experienced robotic surgeons. Analysis of patient and operative characteristics, outcomes, and reasons for abandonment of ORP were described.

**Results:**

Six patients were included for analysis with a median age of 63.5 years [50.3‐67.5]. The median body mass index (BMI) was 34.7 [27.8‐36.2]. All patients had intermediate‐risk prostate cancer. Small prostate and deep pelvis were given as reasons for abandoning ORP in five cases (83.3%), with four of these also attributing increased BMI as a factor. Extensive mesh from previous bilateral inguinal hernia repair was cited as the reason for abandonment in the remaining patient. One patient had commenced androgen deprivation therapy following abandoned ORP. Extensive retropubic adhesions were noted at the time of RARP in five of six patients, with intraoperative complication of small bladder lacerations encountered in the patient with prior mesh hernia repair. The median time from abandoned ORP to RARP was 128 days [40‐216]. Median operating time was 160 minutes [139‐190] and estimated blood loss was 225 mL [138‐375]. Negative margins were obtained in four of six cases, with further salvage treatment being required in one case at a median follow‐up duration of 10.5 months [6.5‐25.3].

**Conclusion:**

Abandonment of ORP is an uncommonly reported event, however, in this small case series, we demonstrate that, in the hands of experienced surgeons, RARP is a safe and technically feasible alternative in such cases. Increased BMI, small prostate size and pelvic anatomical constraints appear to be common catalysts for abandonment of open surgery in this cohort. Identifying these high‐risk patients early and considering referral to robotic centers may be preferred.

## 1 INTRODUCTION

Although modern surgical technique for radical prostatectomy has been well described since the 1980s, it can still present numerous technical challenges to even the most experienced urologist regardless of the technique employed.^1^ In general, these challenges largely relate to accessibility to the prostate gland within the pelvis which can be affected by several patient factors (Box 1).^2^, ^3^, ^4^ Intraoperative abandonment of open retropubic radical prostatectomy (ORP) is an infrequent event with little published literature available. Historically, treatment options following inability to complete surgical resection in this instance would include less invasive techniques such as external beam radiotherapy, brachytherapy, or watchful waiting.^5^ However, with widespread adoption of robotic‐assisted radical prostatectomy (RARP) over the past two decades, significant advances in surgical expertise in this field has improved ability to troubleshoot many of the patient factors that may preclude successful ORP.^6^, ^7^

BOX 1

**Table:** 

Factors impacting surgical difficulty for RP
Patient obesity
Narrow or deep pelvis
Prominent pubis
Extremes of prostate size
History of radiotherapy
Prior pelvic and abdominal surgery

Recently we have been referred a number of patients who have undergone attempted ORP with intraoperative abandonment for various anatomical and patient factors. We were successfully able to perform RARP as a salvage procedure. To the best of our knowledge, there is no preexisting literature examining such techniques. In this case series, we discuss the reasons for abandonment of ORP, technical challenges in approaching such cases, and outline short‐term oncological and functional outcomes of these patients.

## 2 PATIENTS AND METHODS

In a multicenter retrospective review, six patients between 2016 and 2020 were identified to have undergone RARP following abandoned ORP. Two high‐volume robotic surgeons (DGM and DM), who each perform greater than 100 RARPs per year, performed the procedures. Analysis of patient characteristics and their risk factors for difficult radical prostatectomy was undertaken, including reasons for abandonment of ORP. Operative video recordings of subsequent RARP were analyzed and areas of intraoperative difficulty assessed. Postoperative outcomes including histology, hospital length of stay, and complication rate were recorded. Means and standard deviation (±SD) or medians and interquartile ranges [25‐75] were calculated for continuous variables (depending on whether data were para‐ or nonparametric).

### 2.1 Patient characteristics

Demographics, risk factors, and perioperative characteristics at time of primary attempted ORP are outlined in Table 1. All patients were treated for biopsy proven intermediate risk prostate cancer; International Society of Uro‐Pathology (ISUP) grade group 2 in five patients, and ISUP grade group 3 in one patient. Preoperative mean PSA was 6.4 ± 1.9 ng/mL, and clinical stage was T1c in four patients, and T2c in two patients. Four patients were classified as obese (Body Mass Index [BMI] > 30), one was of Afro‐Caribbean descent (known risk factor for a narrow pelvis), and one patient had a past history of bilateral mesh inguinal hernia repair.

**TABLE 1:** Patient characteristics and risk factors for difficult radical prostatectomy

Characteristic	Patients n = 6
Age at surgery (years)	63.5 [50.3‐67.5]
BMI (kg/m)	34.7 [27.8‐36.2]
Preoperative PSA (ng/mL)	6.4 ± 1.9
Preoperative ISUP grade group at biopsy	
ISUP 2	5 (83.3%)
ISUP 3	1 (16.6%)
Pre‐op stage	
pT1c	4 (66.6%)
pT2c	2 (33.3%)
Previous abdominal surgery	1 (16.6%) (Bilateral mesh inguinal hernia repair)
Reasons for abandonment of ORP	
Pelvic anatomical constraints	5 (83.3%)
Elevated BMI (>25)	5 (83.3%)
Prominent pubis	2 (33.3%)
Extensive mesh	1 (16.6%)
Small/impalpable prostate	2 (33.3%)
Alternative therapies prior to RARP	
EBRT	1 (16.6%)
ADT	1 (16.6%)
Time elapsed between abandoned ORP and RARP (days)	128 [40‐216]

Note

Mean ± standard deviation, Median [interquartile range 25‐75].

Abbreviations: ADT, androgen deprivation therapy; BMI, body mass index; EBRT, external beam radiotherapy; ISUP, international society of Uro‐pathology; ORP, open radical prostatectomy; PSA, prostate‐specific antigen; RARP: robot‐assisted radical prostatectomy.

### 2.2 Abandonment of open prostatectomy

In all cases, initial attempted ORP was performed at external hospitals by experienced open surgeons. Reasons for abandonment were as described in operative reports and referral, including combination of elevated BMI, pelvic anatomical constraints and small, impalpable prostate in five cases. Loss of tissue planes due to extensive mesh inguinal hernia repair in a man with an otherwise normal stature and BMI was the cited cause for abandonment in the remaining patient. Median time between abandoned ORP and RARP was 128 days [40‐216]. Only one patient received adjuvant treatment during this intervening time (androgen deprivation therapy (ADT)), however, two patients were initially referred for definitive external beam radiotherapy as an alternative treatment. This was commenced in one patient with initiation of ADT and implantation of fiducial seeds, however, the patient had a history of ulcerative colitis and developed rectal bleeding so radiotherapy was abandoned after only one fraction. Radiotherapy was declined by the second patient, aged 48 years, who sought robotic surgical opinion at our institution instead.

### 2.3 Surgical approach and anatomical challenges

**FIGURE 1:**
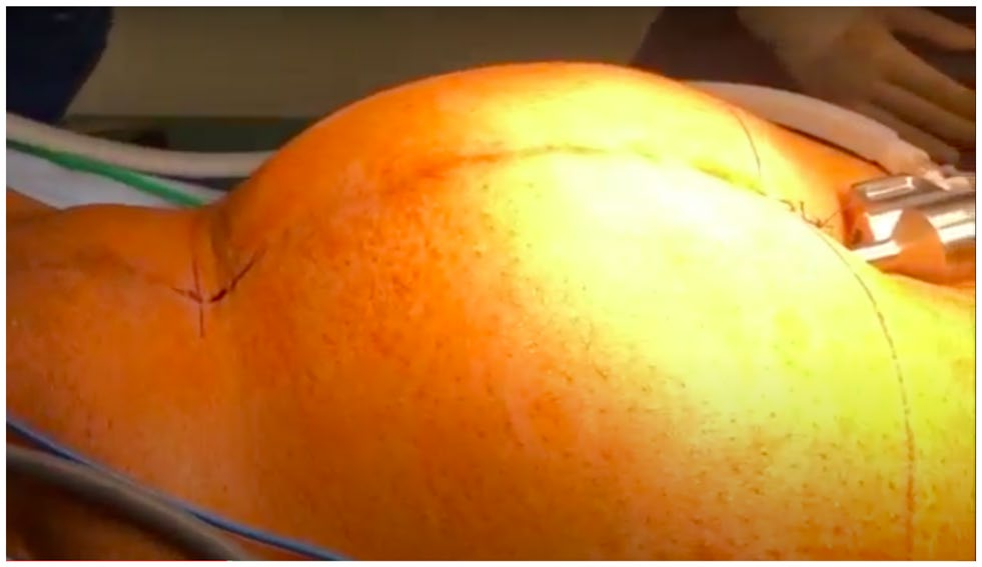
Appearance of insufflated abdomen demonstrating supra‐umbilical Hassan entry. Demonstrates lower midline scar with tethering of the abdominal wall inward

We describe our surgical technique and experience in these cases in the accompanying video. Following routine patient positioning and preparation, open Hassan entry was performed supra‐umbilically avoiding the old lower midline scar (Figure 1). In the case of the patient who underwent RARP only 1 day following abandoned ORP, the lower midline incision was left intact to maintain insufflation. Upon introduction of the camera, the abdomen was carefully inspected for adhesions that could affect port placement. Routine port placement was able to be performed in all cases and instruments introduced.

**FIGURE 2:**
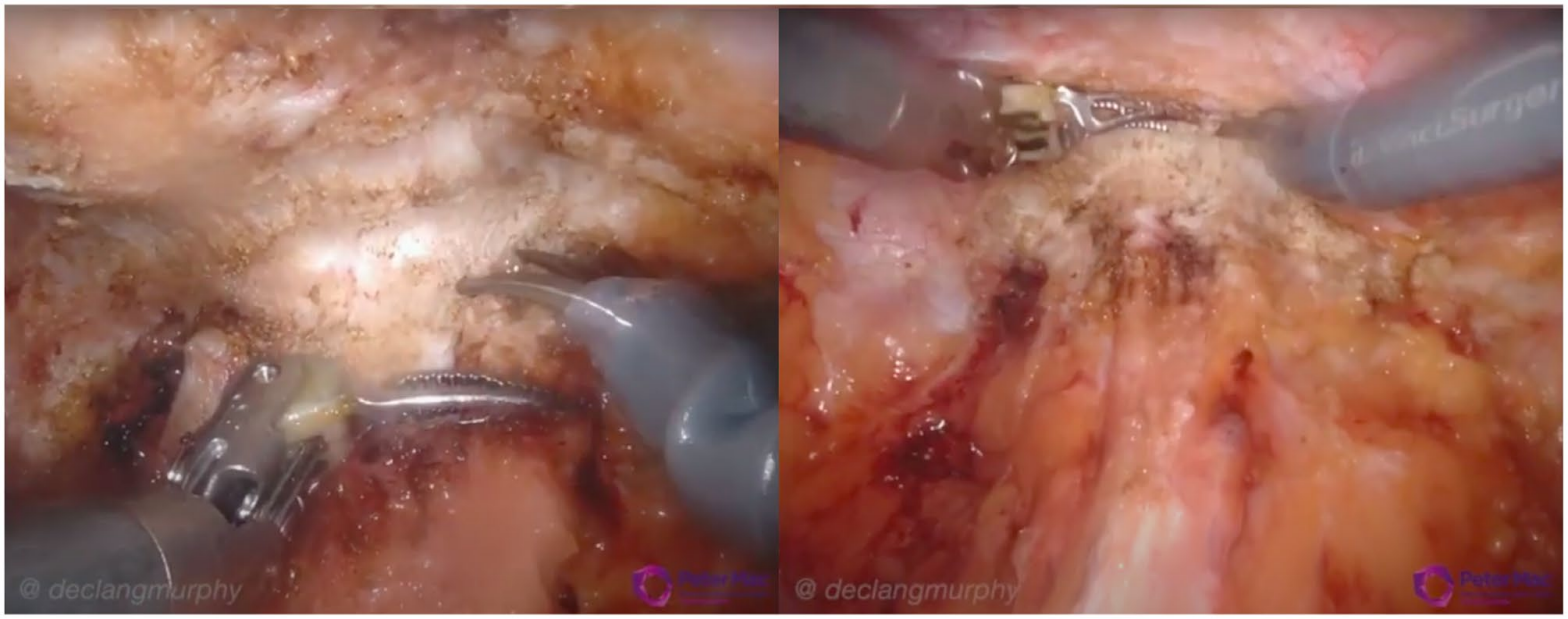
Dissection through dense fibrosis beneath previous lower midline incision down to retropubic space with puckering of the anterior abdominal wall

Commencing with the release of the bladder from the anterior abdominal wall, the presence of dense fibrosis beneath the previous lower midline incision was apparent in the majority of cases, tethering the anterior abdominal wall inward (Figure 2). Of note this scarring was much worse than that experienced, for example, in other parts of the pelvis during salvage RARP following radiation, rather mimicking the appearance of patients with previous SPC or pelvic trauma. Particular difficulty with dissection was faced in the case of previous mesh inguinal hernia repair, with small cystotomies unable to be avoided, however, promptly identified. No other intraoperative complications were encountered. Aiming to enter the retropubic space more superior than usual facilitated easier recognition of the correct tissue plane, however, identification of the pubic symphysis could still prove challenging. The exception to these findings was in the one case performed 1 day following abandoned ORP, where minimal scar tissue was encountered.

**FIGURE 3:**
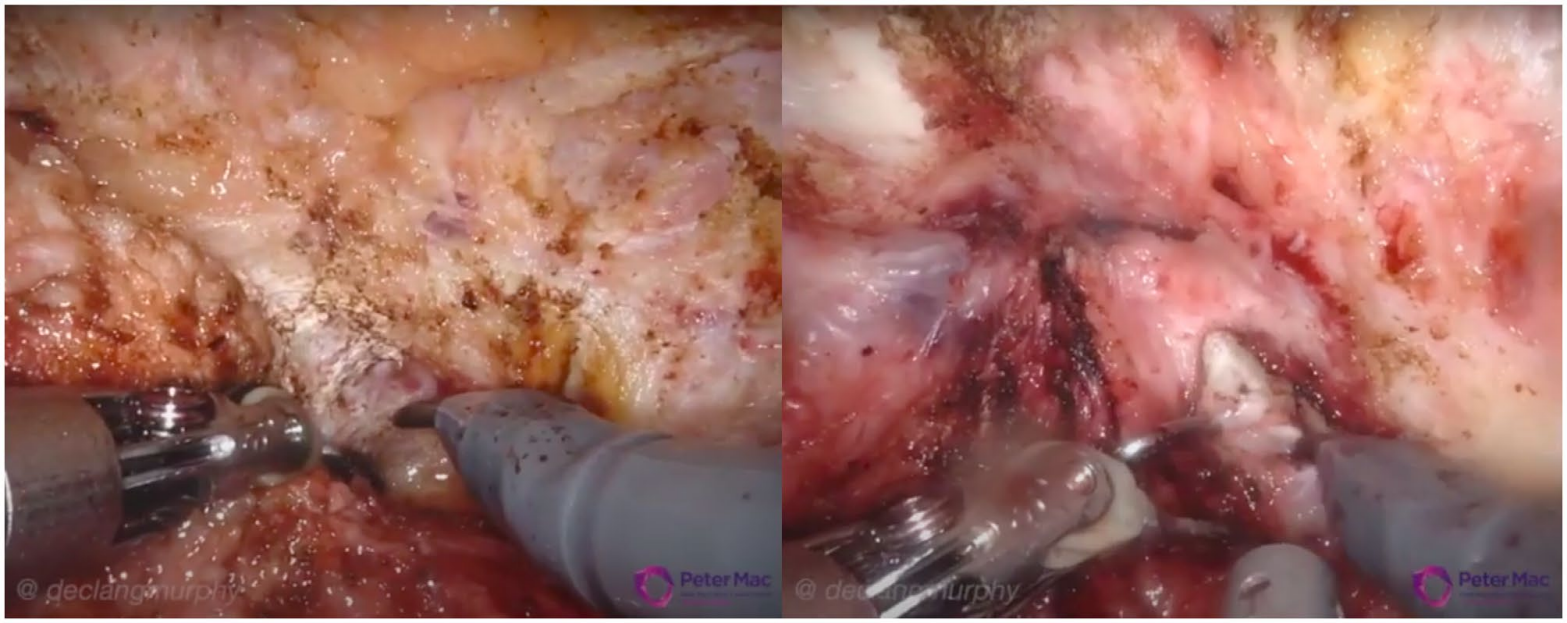
Lateral dissection into the endopelvic fascia with obliteration of natural tissue planes due to prior dissection

Following release of the bladder, lateral dissection identified further fibrous tissue extending into the endopelvic fascia bilaterally in the majority of cases, with the natural tissue planes obliterated (Figure 3). This finding did, however, vary greatly in accordance with the extent of dissection during initial ORP. Care was taken to ensure appropriate planes of dissection were maintained, with particular attention in avoiding bladder injury.

**FIGURE 4:**
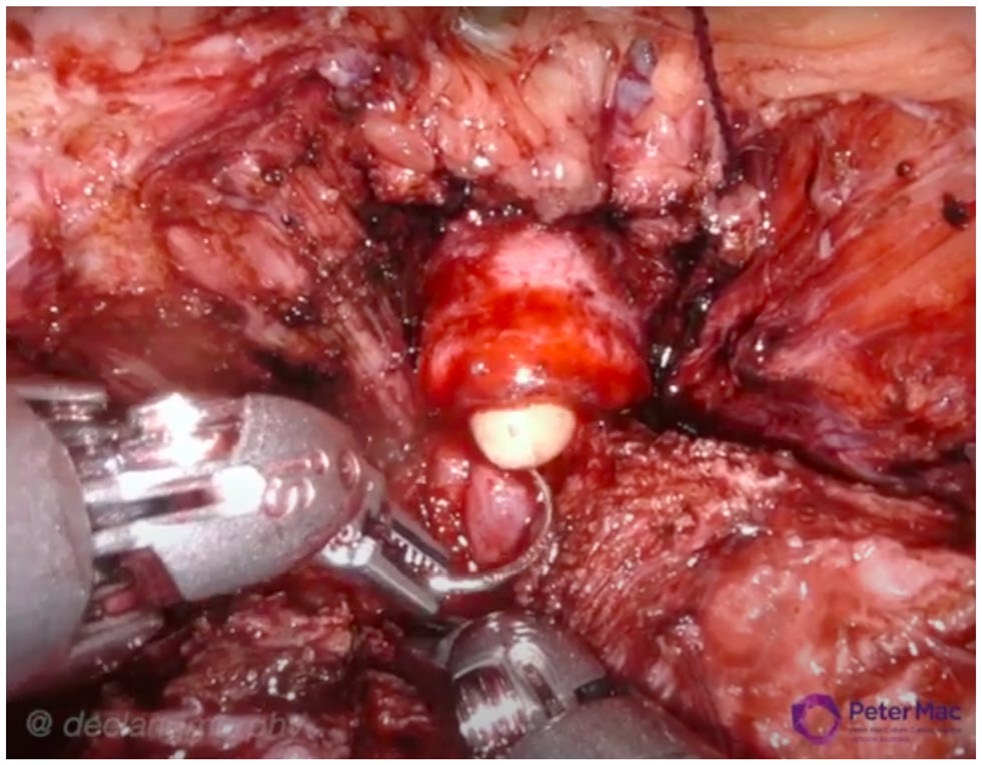
Vesicourethral anastomosis performed largely without difficulty, although in some instance’s mobility restricted by narrow bony pelvis. Good length of urethra obtained

Following dissection of the anterior and posterior bladder neck, seminal vesicles and ligation of the pedicles, attention was turned to further dissection of the endopelvic fascia approaching the apex. Nerve sparing was performed, where safely indicated, using standard athermal retrograde nerve release. In some cases, there was found to be ongoing disruption and scarring from the previous operation in this area. Dorsal venous complex was divided according to surgeon’s routine practice, either following figure of eight suture ligation using Vicryl (n = 2, DM), or cold cut then oversewn with V‐Loc™ (n = 4, DGM). In all cases, however, a good length of urethra was able to be preserved and the anastomosis was performed without any difficulty (Figure 4).

## 3 RESULTS

Perioperative and postoperative outcomes are summarized in Table 2. Each procedure was completed as described with a median operating time, defined as skin incision to closure, of 160 minutes [139‐190] and estimated blood loss of 225 mL [138‐375]. Limited pelvic lymph node dissection was performed in one patient only and mean hospital length of stay was 1.7 ± 0.82 days. Two patients were readmitted within 30 days for Clavien‐Dindo 2 complications; urosepsis and intraabdominal collection of which each were managed with intravenous antibiotics.

**TABLE 2:** Perioperative and postoperative outcomes

Characteristic	Patients n = 6
Operative time (minutes)	160 [139‐190]
Estimated blood loss (mL)	225 [138‐375]
Difficult aspects	
Thickened endopelvic fascia	2 (33.3%)
Adhesions	1 (16.6%)
Mesh	1 (16.6%)
Loss of tissue planes	5 (83.3%)
In Hospital Stay (days)	1.7 ± 0.82
Days postoperatively for IDC removal	10.5 ± 1.38
<30‐day readmission	2 (33%)
Final histopathology ISUP Grade Group	
ISUP 2	1 (16.6%)
ISUP 3	4 (66.7%)
ISUP 5	1 (16.6%)
Margin	
Negative	4 (66.7%)
Positive	2 (33.3%)
Extra‐prostatic extension present	3 (50%)
Surgical specimen prostate size (grams)	42 ± 14.8
PSA to date (ng/mL)	0.044 ± 0.076
3‐month continence	
Fully continent	3 (50%)
Requiring pads	3 (50%)
6‐month continence*	
Fully continent	4 (66.7%)
Requiring pads	1 (16.6%)
Erectile function*	
No erectile function	4 (66.7%)
Functional erectile function	1 (16.6%)
Follow‐up time (months)	10.5 [6.5‐25.3]

Note

Mean ± standard deviation, Median [interquartile range 25‐75].

Abbreviations: IDC, in‐dwelling catheter; ISUP, international society of Uro‐pathology; PSA, prostate‐specific antigen.

* Data missing for one patient due to insufficient follow‐up time.

Upgrading on final pathology was noted in most cases. With the exception being the patient whose RARP was performed only 1 day following abandoned ORP, thereby avoiding time for disease progression after biopsy. Four specimens confirmed ISUP grade group 3 PCa, with grade group 2 and grade group 5 in the remaining two. Specimens had a mean weight of 43 ± 14.8 g, and positive surgical margin was demonstrated in two cases (right posterior and anterior base, respectively). Patients with positive margins were each managed with close PSA surveillance, each currently with undetectable PSA at 8‐ and 28‐months. At a median follow‐up time of 10.5 months [6.5‐25.3], only one patient has demonstrated evidence of biochemical recurrence with a PSA of 0.18 ng/ml, negative PSMA‐PET/CT and is undergoing salvage radiotherapy and ADT. In terms of functional outcomes, four patients were fully continent at 6‐months, one requiring one pad/day and the remaining patient had insufficient follow‐up time. Erectile function is absent in five patients at latest follow‐up, with one patient having functional erections without pharmacotherapy (at 24 months follow‐up).

## 4 DISCUSSION

Radical prostatectomy can be a challenging operation whether it be by open, laparoscopic, or robotic‐assisted surgical technique. In the early years of the development of RARP, conversion to open surgery due to complication was reported to be as high as 17%, however, over time has fallen to less than 0.07%.^8^ This is primarily a result of widespread adoption of RARP over the past two decades and considerable advances in surgical expertise for the minimally invasive technique, consequently resulting in proportional reduction in ORP being performed.^6^ In Australia, access to RARP is readily available in the private sector, however, only few, more centralized, public hospitals have access to robotic equipment, including our own institution.^6^ Accordingly, ORP remains readily performed in the public healthcare system. Although intraoperative abandonment of ORP is an uncommon occurrence, traditionally, subsequent treatment would be limited to radiation or non‐curative management in the form of watchful waiting and hormonal therapy.^5^ The shift in surgical proficiency in techniques for radical prostatectomy, however, has seemingly resulted in an inverse turn of events whereby abandonment of ORP may successfully be managed with conversion to RARP as demonstrated in our case series.

Surgical difficulty in performing radical prostatectomy can be attributed to both patient and anatomical factors. Patient ethnicity, pelvic dimensions, and BMI have all been associated with prolonged operative time, and increased rates of positive surgical margin regardless of approach.^9^, ^10^ Increased BMI results in decreased working space within the pelvis due to periprostatic and peri‐vesical fat. Obese patients may have a caudally displaced pelvic floor due to increased intraabdominal weight, resulting in increased distance between the bladder neck and membranous urethra, and thus, a technically challenging vesicourethral anastomosis.^2^, ^3^ Extremes of prostate size in each direction appear to impact surgical difficulty, with large prostates being associated with longer operative time and higher estimated blood loss, and small prostates having increased risk of positive margin.^4^ Traditionally, large prostate volume is documented to be a contributing factor to difficult RP, however, interestingly in our experience two of the six patients were referred with small or impalpable prostates being the contributing cause for abandonment. Reasons for this may be explained by a cumulative effect of a small prostate in an obese patient and narrow pelvis ultimately resulting in inability to safely gain vision and anatomical control. Operative difficulty due to prior laparoscopic inguinal hernia repair is well documented, with anecdotal cases resulting in abandonment of surgery.^11^ Localized inflammatory reaction following inguinal hernia repair can result in obliteration of the retropubic space, impairment of retraction, and increased risk of complications.^3^, ^11^ This is further highlighted in our experience by the intraoperative complication of bladder injury in the patient with prior mesh inguinal hernia repair. Two of our patients were readmitted with complications less than 30‐days port‐operatively. We believe this reflects the increased complexity of reoperating after previous surgery in this area, yet, were only short‐term complications managed medically and did not influence long‐term outcomes.

Similarities regarding destruction of natural tissue planes and inflammatory changes can be drawn from salvage RARP for biochemical and localized recurrence following EBRT for PCa. Previously reserved for open technique, salvage prostatectomy by robotic approach has been shown to offer equivalent oncological outcomes with shorter convalescence.^12^, ^13^ Some authors have proposed that RARP is often preferable to open and laparoscopic techniques for salvage and complex prostate surgery due to improved visualization and mobility, allowing easier identification of surgical planes and optimization of functional outcomes by superior neurovascular protection.^14^, ^15^ Our experience suggests that RARP following abandoned ORP often results in similarly troublesome scarring and fibrosis of tissue, however, when compared to salvage RARP following EBRT, this is more so present anteriorly and apically with the posterior dissection largely unaffected. Surgeons performing difficult ORP should be cognizant that abandoning the procedure early will allow preservation of tissue planes in subsequent steps of the operation in the event of considering RARP as alternative approach. Furthermore, prompt reoperation, in a matter of days, was shown to be preferable in order to avoid difficulty with adhesions entirely as well as reduce risk of upstaging of pathology due to delay in treatment.

This study has limitations due to its small sample size, retrospective analysis of prospectively collected data, and potential selection bias. Nonetheless, we provide a pertinent discussion of the evolving role of RARP in the event of inability to perform ORP. Whether there has been some erosion of open surgical skills due to the reduction in ORP volume in public hospitals is not clear, and it is not known if cases such as these reported here might have been successfully concluded if done in a different era by more experienced surgeons. Either way, it appears that intraoperative abandonment of ORP is a reality in the contemporary era, and salvage options such as RARP need to be considered. Upon evaluation of safety, feasibility, oncological, and continence outcomes, we obtained favorable results in this patient group.

## 5 CONCLUSION

RARP can successfully overcome constraints encountered during ORP, and should be considered as an alternative management in the event of abandoned ORP. Pelvic anatomy, obesity, and prior inguinal mesh hernia repair should be considered preoperatively as potential risks factors for difficult ORP. In the event of ORP abandonment, prompt referral to robotic centers for timely reoperation is encouraged to avoid development of tissue fibrosis and obliteration of operative planes.

## CONFLICT OF INTEREST

All authors have no conflicts of interest or financial disclosures to be made

## AUTHOR CONTRIBUTIONS

E. O’Connor, S. Koschel, D. Bagguley—writing, data curation & analysis; D. Moon, D. G. Murphy—conceptualization, methodology, critical review & editing; I. A. Thangasamy, N. J. Sathianathen, M. G. Cumberbatch—methodology, critical review & editing.
